# Adherence to food-based dietary guidelines among adolescents in Germany according to socio-economic status and region: results from Eating Study as a KiGGS Module (EsKiMo) II

**DOI:** 10.1017/S136898002100001X

**Published:** 2021-04

**Authors:** Anna-Kristin Brettschneider, Clarissa Lage Barbosa, Marjolein Haftenberger, Franziska Lehmann, Gert BM Mensink

**Affiliations:** Department of Epidemiology and Health Monitoring, Robert Koch Institute, General-Pape-Str. 62-66, Berlin 12101, Germany

**Keywords:** Food consumption, Food-based dietary guidelines, Adolescents, Germany, National nutrition survey

## Abstract

**Objective:**

Dietary habits developed during childhood and adolescence are likely to continue into adulthood. An unbalanced diet may cause nutrient deficiencies and excessive energy intake; these enhance the risk for developing overweight and obesity and their co-morbidities. In the present analysis, food consumption of adolescents is described and evaluated against German food-based dietary guidelines with special focus on socio-economic status (SES) and region of residence.

**Design:**

Within the ‘German Health Interview and Examination Survey for Children and Adolescents’ (KiGGS Wave 2), the cross-sectional ‘Eating Study as a KiGGS Module’ (EsKiMo II) was conducted from 2015 until 2017 to provide data about dietary behaviour.

**Setting:**

Germany.

**Participants:**

1353 adolescents aged 12–17 years from a nationwide representative sample with food consumption data from computer-assisted dietary history interviews.

**Results:**

The median consumption of fruits, vegetables, starchy foods and milk/dairy products among adolescents in Germany was below the recommendation. The median consumption of both meat/meat products and unfavourable foods, like confectionery, which should be consumed sparingly, was about 1·5 times the recommended amount. The total amount of beverages consumed by most adolescents was above the minimum amount recommended. Soft drink consumption of adolescents with a low SES was three to five times higher than soft drink consumption of adolescents with a high SES.

**Conclusions:**

The results indicate the need for an improvement of dietary habits among adolescents in Germany. Further approaches to promote healthy diets in Germany should be continued, and the focus on social inequalities should be strengthened.

There are many known relationships between diet and health outcomes^(1)^. An unbalanced diet may cause nutrient deficiencies and excessive energy intake which can increase the risk of becoming overweight or obese. Being overweight or obese is a risk factor for various diseases such as diabetes mellitus type 2 or CVD. In addition, being overweight or obese in childhood is very likely to be continued into adulthood^(2)^. Taste preferences and dietary habits developed during childhood may also last for a lifetime^(3,4)^. Associations between social disparities and health behaviour among adolescents have been observed; a lower socio-economic status (SES) is associated with less participation in sports, poorer diets and higher prevalence rates for being overweight and obese^(5–7)^. International and national research focusing on dietary behaviour shows that there is lower consumption of fruit^(8,9)^ and vegetables^(8–10)^ among adolescents with a low SES compared with adolescents with a high SES. Among adolescents with a low SES, the consumption of sugar-sweetened beverages^(8,11–13)^ and fast food^(10,14)^ is higher than that of their counterparts with a high SES.

Food consumption habits are changing continuously due to changes in the food supply and other reasons that include an extension of the range of products as well as changes in living conditions. For example, the all-day school system in Germany has been expanded in the last years. Therefore, school meals have an increasing influence on food consumption among children and adolescents^(15)^. In former East Germany, all-day schools were more common than in former West Germany; therefore, the utilisation of school meals among children and adolescents was also more common^(15)^. Therefore, it is still interesting to compare food consumption in former East and West Germany almost 30 years after the reunification.

National representative dietary surveys provide relevant information on food consumption of the general population and specific groups. Such information, including deficits in food consumption in specific population groups, is needed to identify relevant fields of action for public health interventions. This is relevant for decision-makers in the field of public health nutrition. The ‘German Health Interview and Examination Survey for Children and Adolescents’ (KiGGS) is part of the national health monitoring system of the Robert Koch Institute^(16)^. Within the KiGGS baseline survey (2003–2006), a study with in-depth focus on nutrition, the ‘Eating Study as a KiGGS Module’ (EsKiMo), was conducted in 2006^(17)^. About a decade later, EsKiMo II (2015–2017) was conducted as part of KiGGS Wave 2. This newly available information about food consumption of children and adolescents living in Germany may help to identify deficits and excesses in food consumption and risk groups^(18,19)^. The aim of this analysis is to describe the food consumption among 12- to 17-year-old adolescents in general and to stratify it by SES as well as by the region of residence and to evaluate their food consumption in relation to German food-based dietary guidelines according to the concept of the Optimized Mixed Diet (OMD)^(20)^.

## Methods

### Study design

EsKiMo II was implemented within the second wave of KiGGS – KiGGS Wave 2 and conducted between 2015 and 2017 with the aim of providing representative data on food consumption and nutritional status among children and adolescents living in Germany. For EsKiMo II, an age- and sex-stratified sample of 2644 children and adolescents aged 6–17 years was drawn from the study sample of the cross-sectional part from KiGGS Wave 2. Since different instruments were used to assess food consumption depending on participants’ age^(18,19)^, the present analysis is restricted to 1353 adolescents aged from 12 to 17 years.

### Dietary assessment

Participants aged from 12 to 17 years were interviewed about their food consumption during the past 4 weeks using the software DISHES (Dietary Interview Software for Health Examination Studies) which is based on a modified dietary history method developed by the Robert Koch Institute^(17,21)^. The interviews were conducted face-to-face by trained nutritionists during home visits. Following a daily meal structure (breakfast, mid-morning snack, lunch, mid-afternoon snack, dinner, small late meal and snacks throughout the day), the consumption frequencies and portion sizes of all foods consumed were recorded. Tableware models and a picture book of portion sizes^(22,23)^ were used to assist in the estimation of portions. A validation study among adults showed that differences in macronutrient intake between DISHES and 3-d dietary records and 24-h recalls were within an acceptable range for assessing food consumption in epidemiological studies^(21)^. Estimation of food consumption using DISHES showed fairly good agreement with a FFQ among children^(24)^. For the selection of food items, the German Nutrient Data Base (Bundeslebensmittelschlüssel, Max Rubner Institute) Version 3.02 was integrated in the DISHES software.

### Food group quantities

Quantities were calculated on the basis of the amount of foods as they were consumed (e.g. cooked, fried, raw). Composed dishes, which could not be unequivocally assigned to one food group, were decomposed into their ingredients, and these were assigned to the corresponding food group. The mean daily amount of each food consumed was obtained by multiplying the consumption frequency by the portion amount and dividing this by 28 (4 weeks ≙ 28 d). The daily amounts consumed were summarised in food groups. For solid foods, fourteen different groups (bread, savoury baked goods; grain, pasta, rice; breakfast cereals; salty snacks; vegetables; fruits; potatoes; eggs; fats and oils; milk/dairy products, cheese, curd; meat/meat products; fish; confectionery, cake; other food items) and for beverages, six groups (tea; coffee; juice; soft drinks; alcoholic beverages; water) were constructed. Table 1 gives an overview of the food groups.

**Table 1:** Description of the analysed food groups

Main group	Examples
Bread, savoury baked goods	Bread, bread rolls, bread products, savoury baked goods
Grain, pasta, rice	Grain, flours, grain products, rice, noodles
Breakfast cereals	Cornflakes, muesli, cereals
Salty snacks	Crisps, crackers and salted sticks
Vegetables	Cooked and raw vegetables (excluding potatoes), legumes, vegetable products
Fruits	Cooked and raw fruits, fruit products
Potatoes	Potatoes and potato products
Eggs	
Fats and oils	Butter, lard, margarine, plant oils
Milk/dairy products, cheese, curd	Milk, dairy products, cheese, cream cheese, curd
Meat/meat products	Poultry, meat, offal, mollusc, raw, cooked and pre-cooked sausage
Fish	Fatty fish, lean fish, crustaceans and shellfish
Confectionery, cake	Sugar, sweetener, sweet spreads, sweets, ice cream, chocolate, pralines, confectionery products, desserts, sweet sauces, cakes, biscuits, waffles
Tea	Green tea, black tea, herbal tea, fruit tea
Coffee	Coffee, coffee-based drinks, coffee substitutes
Juice	Fruit and vegetable juice, fruit-based drinks, nectar, smoothies
Soft drinks	Sugar-sweetened beverages, low calorie drinks, isotonic drinks, non-alcoholic beer
Alcoholic beverages	Beer, wine, liquor
Water	Sparkling water, tap water, water as ingredient

### Food-based dietary guidelines for children and adolescents in Germany

The food consumption in EsKiMo II was compared with the OMD recommendations of 2017^(20)^. Within the concept of OMD, the German nutrient recommendations (D-A-CH reference values) were translated into sex- and age-specific food-based dietary guidelines for children and adolescents^(20)^. The recommended age-specific amounts were derived for eleven food groups, considering an energy requirement for a low level of physical activity (PAL 1.4)^(20)^. The foods reported in EsKiMo II were summarised into the eleven OMD food groups. These can generally be categorised in three recommended consumption amount groups:

*foods to be consumed plenty* – ‘beverages’, ‘vegetables’, ‘fruits’, ‘bread/(breakfast) cereals’, ‘potatoes/pasta/rice’;
*foods to be consumed moderately* – ‘milk/dairy products’, ‘meat/meat products’, ‘eggs’, ‘fish’;
*foods to be consumed sparingly* - ‘oil/margarine/butter’, ‘unfavourable foods’ (sweets, pastries, sugar-sweetened beverages).

In order to evaluate food group consumption according to the concept of the OMD, for each individual, we divided the particular food group consumption by the corresponding age- and sex-specific recommendations and multiplied this by 100. The distribution characteristics of these proportion values are presented. For the present analysis, the food groups ‘bread/(breakfast) cereals’ and ‘potatoes/pasta/rice’ were summarised as ‘starchy foods’ (referring to foods with high content of complex carbohydrates). For comparison with the OMD, sugar-containing beverages were included in the food group beverages. In addition, the energies obtained from sugar-containing beverages were included in the group ‘unfavourable foods’. Age- and sex-specific OMD-recommended quantities for adolescents aged 12–17 years are shown in Table 2.

**Table 2:** Recommendations for food consumption quantities according to the food-based dietary guidelines^(20)^

Age	12 years	13–14 years		15–17 years	
		Girls	Boys	Girls	Boys
Foods to be consumed plenty					
Beverages (ml/d)	950	1000	1200	1100	1400
Vegetables (g/d)	300	320	390	340	440
Fruits (g/d)	280	300	360	310	410
Bread/(breakfast) cereals (g/d)	180	190	230	200	260
Potatoes/rice/pasta (g/d)	160	170	200	180	230
Foods to be consumed moderately					
Milk/dairy products* (g/d)	470	490	600	520	680
Meat/meat products (g/d)	50	50	60	50	70
Eggs (pieces/week)	2–3	3	3	3	3–4
Fish (g/week)	90	100	110	100	130
Foods to be consumed sparingly					
Oil/margarine/butter (g/d)	30	30	35	30	40
Unfavourable foods (max. kcal/d†)	180	200	230	190	260

* Milk equivalents, i.e. 100 g milk corresponds to 100 g yogurt or 30 g cheese.

† 100 kcal corresponds to about 20 g chocolate or 30 g jam or 45 g fruit cake or 10 chips or 200 ml lemonade.

### Socio-economic status and region of residence

In KiGGS Wave 2, comprehensive information from participants and their parents was collected. A multidimensional SES index was derived from information provided by the parents about their level of education, occupational status and net income. Therefore, point scores were assigned according to the level of each status dimension ranging from 1 to a maximum of 7, and the sum of these scores was calculated. The three equally weighted subscales of education, occupational status and income provide the basis for calculating the SES index, which means that SES index values ranged between 3·0 and 21·0. This SES index was divided into quintiles and classified into (1) low SES (lowest quintile), (2) medium SES (middle three quintiles) and (3) high SES (upper quintile)^(25)^. Information about the region of residence was classified into (1) former West Germany and (2) former East Germany (including Berlin).

### Statistical analyses

Mean, median, 5th and 95th percentiles for food and beverage consumption in g/d were described separately for girls and boys. Distributions of individual consumption in proportion to the OMD-recommended amounts are presented using Box–Whisker plots with the inner box corresponding to the interquartile range around the median and the whiskers corresponding to the 5th and 95th percentiles.

Mean values with 95 % CI for absolute amounts of food consumption in g/d as well as the proportion of adolescents who achieved the OMD-recommended amounts (%) were calculated in total and for categories of SES and region of residence. Differences in mean food group consumption by SES and region were tested using ANOVA. Differences in the proportion of adolescents who achieved the OMD-recommended amounts by SES and region were tested with the *χ*^2^ test.

To correct for deviations from the German population with regard to age (in years), sex, federal state (as of 31 December 2015), nationality (as of 31 December 2014) and parents’ level of education (Microcensus 2013), as well as for differences in participation in EsKiMo II by parental SES, school type visited by the child and season, a weighting factor was applied in EsKiMo II. To take the cluster design of the study sample into account, the analyses were conducted using the survey procedures of SAS version 9.4 (SAS Institute Inc.). All analyses were stratified by sex.

## Results

The present analysis included 727 girls and 626 boys. Twenty-three percent of the girls and 16 % of the boys lived in families with a low SES and 14 % of the girls and 21 % of the boys in families with a high SES. One-sixth of the adolescents were from former Eastern Germany (Table 3).

**Table 3:** Basic characteristics of the study population of Eating Study as a KiGGS Module (EsKiMo) II (*n* 1353)

	Girls		Boys	
	*n*	%	*n*	%
	727	48·6	626	51·4
Socio-economic status				
Low	76	22·7	53	16
Medium	473	63·6	390	63·2
High	167	13·7	175	20·9
Region of residence				
Former East Germany	249	16·2	216	16·4
Former West Germany	478	83·8	410	83·6

Generally, mean and median food group consumption was higher among boys than girls (Table 4). The mean consumption of beverages among girls amounted to approximately 1700 ml/d, whereas the mean consumption among boys was more than 2000 ml/d. The main beverage was water which contributed to two-thirds of the total beverage consumption of girls and boys. Meanwhile, the contribution of soft drinks, juices and alcoholic beverages to total beverage consumption was about one quarter among girls and one-third among boys. Median consumption of meat/meat products among girls was 74 g/d, whereas among boys, it was 114 g/d. Only the consumption of vegetables, fruits and tea was higher among girls than boys. Median consumption for fruits was 121 g/d and for vegetables 167 g/d among girls and 98 g/d and 142 g/d, respectively, among boys.

**Table 4:** Mean and distribution characteristics of total energy (kcal/d) and food consumption (g/d) among adolescents aged from 12 to 17 years from Eating Study as a KiGGS Module (EsKiMo) II, stratified by sex (*n* 1353)*

	Girls (*n* 727)				Boys (*n* 626)			
	Mean	Median	P5	P95	Mean	Median	P5	P95
Total energy intake (kcal/d)	1866	1780	855	3315	2416	2221	1189	3976
Total food (except beverages) (g/d)	1119	1052	522	1866	1340	1244	679	2323
Bread, savoury baked goods (g/d)	135	122	38	284	160	145	47	318
Grain, pasta, rice (g/d)	82	69	20	180	103	77	27	247
Breakfast cereals (g/d)	11	3	0	47	21	5	0	91
Salty snacks (g/d)	6	3	0	27	7	3	0	26
Vegetables (g/d)	208	167	39	495	178	142	33	411
Fruits (g/d)	163	121	13	407	136	98	5	410
Potatoes (g/d)	65	53	8	166	83	65	11	208
Eggs (g/d)	15	11	0	48	21	14	2	59
Fats and oils (g/d)	24	21	6	54	30	24	6	76
Milk/dairy products, cheese, curd (g/d)	201	164	23	516	318	241	23	863
Meat/meat products (g/d)	87	74	3	218	130	114	24	280
Fish (g/d)	9	4	0	31	13	6	0	43
Confectionery, cake (g/d)	90	70	10	229	112	90	14	264
Total beverages (g/d)	1727	1590	642	3141	2035	1887	932	3612
Tea (g/d)	152	32	0	643	81	0	0	343
Coffee (g/d)	26	0	0	182	29	0	0	214
Juice (g/d)	177	77	0	671	243	115	0	879
Soft drinks (g/d)	201	51	0	944	352	143	0	1500
Alcoholic beverages (g/d)	22	0	0	118	46	0	0	317
Water (g/d)	1150	1033	114	2516	1284	1143	100	2954

P5, 5th percentile; P95, 95th percentile.

* Weighted figures except *n* (= unweighted sample number).

**Fig. 1:**
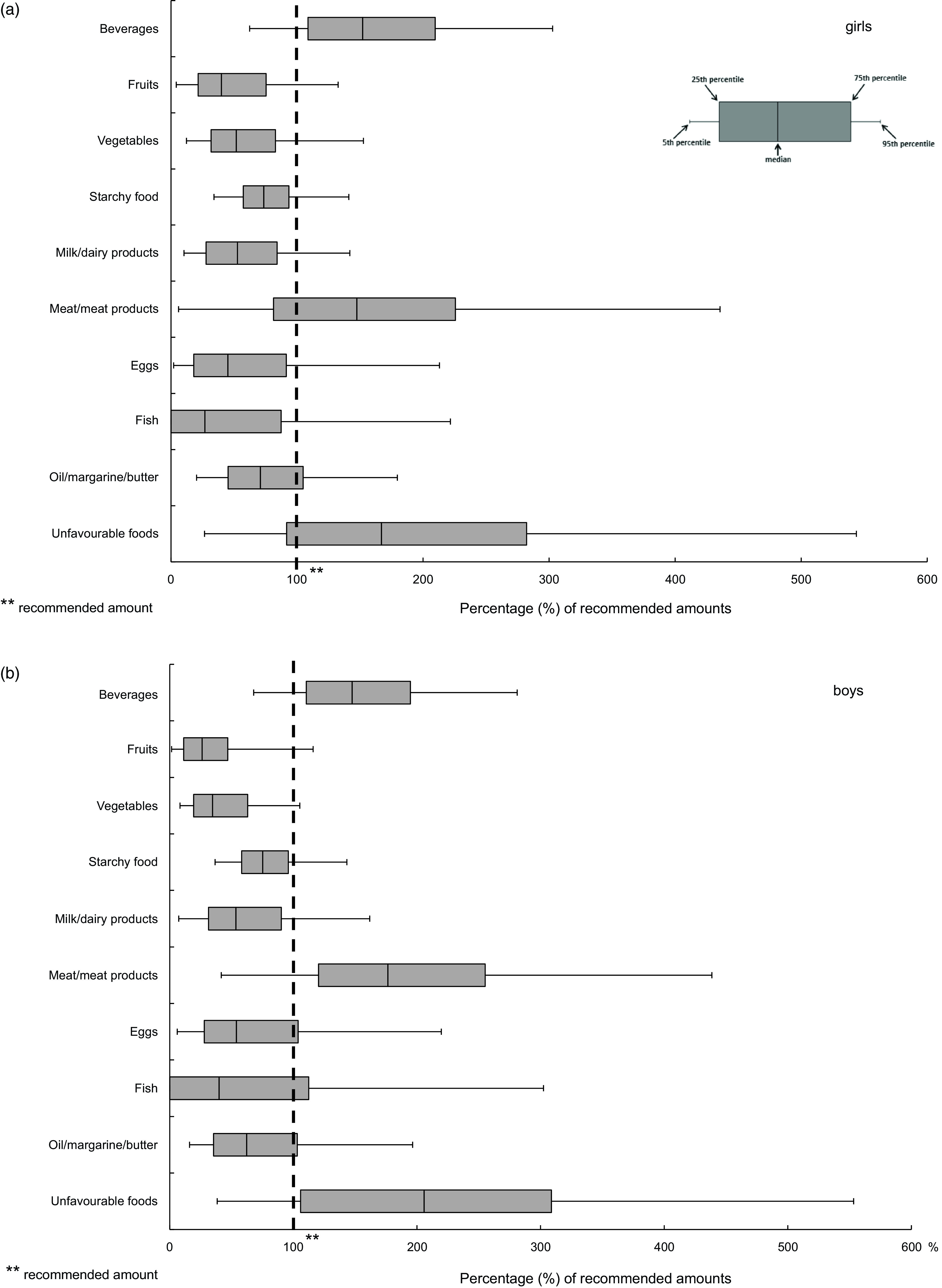
Distribution of food consumption expressed as the proportion of the recommended amounts for an OMD among 12- to 17-year-old (a) girls and (b) boys in EsKiMo II (*n* 1353) (Box–Whisker plots*) * Box–Whisker plots with the inner box corresponding to the interquartile range around the median and the whiskers corresponding to the 5th and 95th percentiles

Figure 1 shows the distribution (using Box–Whisker plots) of food group consumption among girls and boys expressed as proportions of the OMD-recommended amounts. The total amount of beverages consumed by most of the adolescents was above the minimum amount recommended. The median consumption of fruits, vegetables, starchy foods, milk/dairy products, eggs, fish and oil/margarine/butter of adolescents was below the recommended amounts. This indicates that more than half of the adolescents did not achieve the recommendations for these food groups. For meat/meat products and unfavourable foods, the median consumption was far above the recommended amounts. More than half of the boys had a consumption of unfavourable foods twice as high as the recommended amounts (Fig. 1).

A comparison of the mean consumption of food groups by the SES of the family is presented in Table 5. Girls with a low SES consumed on average more meat/meat products than girls with a high SES (103 g/d *v*. 67 g/d; *P* < 0·01). The mean consumption of soft drinks was five times higher among girls with a low SES than those with a high SES (314 g/d *v*. 62 g/d; *P* < 0·001). Among boys, it was three times higher for those with a low SES compared with their counterparts with a high SES (587 g/d *v*. 188 g/d; *P* < 0·001). Meanwhile, boys with a high SES had a higher juice consumption than boys with a low SES (155 g/d *v*. 254 g/d; *P* < 0·05).

**Table 5:** Mean and 95 % CI of food consumption (g/d) among adolescents from Eating Study as a KiGGS Module (EsKiMo) II by sex and socio-economic status (SES) (*n* 1334)*

	Girls (*n* 716)							Boys (*n* 618)						
	SES							SES						
	Low (*n* 76)		Medium (*n* 473)		High (*n* 167)			Low (*n* 53)		Medium (*n* 390)		High (*n* 175)		
	Mean	95 % CI	Mean	95 % CI	Mean	95 % CI	*P* †	Mean	95 % CI	Mean	95 % CI	Mean	95 % CI	*P* †
Total foods (except beverages)	1128	996, 1259	1124	1072, 1176	1087	1000, 1173	0·771	1338	1127, 1549	1304	1247, 1360	1452	1325, 1578	0·114
Bread, savoury baked goods	130	105, 155	134	124, 144	139	124, 155	0·798	157	130, 184	157	148, 167	171	153, 188	0·367
Grain, pasta, rice	81	64, 97	83	75, 90	87	76, 98	0·789	88	66, 110	97	88, 106	124	98, 149	0·070
Breakfast cereals	9	5, 13	12	9, 14	15	11, 19	0·170	12	-1, 24	23	15, 31	23	18, 28	0·205
Salty snacks	7	5, 10	6	5, 8	4	3, 6	0·061	5	1, 9	7	6, 9	7	5, 8	0·622
Vegetables	234	184, 284	197	182, 213	213	185, 241	0·252	157	120, 194	176	157, 195	197	166, 228	0·281
Fruits	141	105, 176	171	156, 186	167	134, 200	0·287	157	89, 225	121	107, 134	163	131, 195	0·040
Potatoes	67	53, 80	67	60, 73	56	49, 62	0·049	88	49, 128	83	74, 92	78	68, 88	0·732
Eggs	18	13, 23	15	13, 17	13	10, 16	0·177	27	19, 35	21	18, 24	18	15, 21	0·038
Fats and oils	24	21, 27	25	23, 27	23	20, 27	0·776	28	21, 35	30	27, 32	31	27, 34	0·773
Milk/dairy products, cheese, curd	199	158, 241	202	180, 224	195	168, 223	0·923	335	226, 443	305	270, 340	359	278, 440	0·443
Meat/meat products	103	82, 124	86	78, 95	67	56, 79	0·003	149	112, 186	127	117, 137	127	114, 139	0·497
Fish	8	5, 12	9	7, 10	9	6, 12	0·921	12	2, 22	13	10, 15	12	10, 15	0·924
Confectionery, cake	83	62, 105	96	87, 104	80	67, 93	0·091	91	71, 110	117	108, 127	117	104, 131	0·041
Total beverages	1783	1605, 1962	1726	1627, 1825	1676	1427, 1925	0·746	2013	1723, 2303	2095	1974, 2216	1851	1694, 2009	0·054
Tea	138	78, 199	161	117, 205	126	89, 164	0·514	70	26, 114	79	53, 106	80	49, 112	0·929
Coffee	30	5, 56	25	15, 34	25	1, 49	0·922	37	9, 66	28	18, 37	28	15, 41	0·808
Juice	185	115, 255	175	141, 210	172	106, 239	0·956	155	88, 223	260	211, 310	254	199, 308	0·030
Soft drinks	314	198, 431	194	155, 233	62	46, 79	< 0·001	587	385, 789	352	285, 418	188	141, 236	< 0·001
Alcoholic beverages	11	4, 18	28	10, 46	15	6, 23	0·215	39	5, 72	52	35, 68	40	20, 59	0·572
Water	1104	877, 1331	1143	1052, 1234	1275	1037, 1514	0·512	1124	814, 1435	1325	1209, 1440	1261	1090, 1433	0·440

* Weighted figures except *n* (= unweighted sample number), number of adolescents with missing SES: *n* 19.

† Significant differences between groups were tested using ANOVA.

For most food groups, there were no statistical differences among SES groups regarding the proportion achieving the OMD-recommended amounts (Table 6). However, the proportion of boys who achieved the recommended amounts of fruit differed significantly by SES. Boys with a low SES met the recommended amount of fruit more often compared with boys with a medium or high SES (*P* < 0·05).

**Table 6:** Proportion of adolescents from Eating Study as a KiGGS Module (EsKiMo) II achieving the Optimized Mixed Diet (OMD)-recommended amount by sex and socio-economic status (SES) (*n* 1334)*

	Girls (*n* 716)							Boys (*n* 618)						
	SES							SES						
	Low (*n* 76)		Medium (*n* 473)		High (*n* 167)			Low (*n* 53)		Medium (*n* 390)		High (*n* 175)		
	%	95 % CI	%	95 % CI	%	95 % CI	*P* †	%	95 % CI	%	95 % CI	%	95 % CI	*P* †
Fruits	17·4	6·3, 28·8	15·2	11·1, 19·3	12·8	7·3, 18·3	0·773	12·7	2·7, 22·7	4·0	1·9, 6·0	9·3	3·4, 15·2	0·017
Vegetables	22·8	10·8, 34·9	14·5	10·7, 18·3	20·6	11·7, 29·6	0·203	8·5	0·0, 17·1	6·9	3·6, 10·3	7·4	3·2, 11·6	0·915
Starchy food	16·9	7·6, 26·2	21·7	15·9, 27·4	24·1	16·9, 31·2	0·520	19·7	5·8, 33·5	18·6	13·9, 23·2	20·5	13·7, 27·3	0·937
Meat/meat products	68·9	54·9, 82·8	67·2	61·6, 72·7	54·2	44·4, 64·0	0·218	87·5	76·0, 99·0	82·1	77·3, 86·9	81·0	74·3, 87·6	0·592
Fish	20·5	9·5, 31·3	20·5	16·2, 24·8	19·4	10·6, 28·1	0·984	20·0	6·4, 33·6	30·3	24·5, 36·1	33·0	24·7, 41·4	0·271
Eggs	25·5	14·2, 36·6	22·8	17·4, 28·3	15·8	8·7, 22·9	0·387	37·1	19·6, 54·7	25·0	19·4, 30·6	19·2	11·6, 26·9	0·065
Milk/dairy products	13·7	5·2, 22·1	17·6	12·6, 22·5	14·5	8·3, 20·6	0·608	19·5	5·4, 33·7	18·6	13·8, 23·3	21·2	13·7, 28·6	0·892
Oil/margarine/butter	25·0	13·0, 36·2	30·0	24·9, 35·2	28·9	20·8, 37·2	0·657	21·7	9·7, 33·7	25·7	20·4, 31·0	28·1	20·4, 35·8	0·686
Beverages	84·1	75·3, 92·9	80·8	75·6, 86·0	74·8	64·4, 85·1	0·719	84·0	71·7, 96·2	83·0	77·8, 88·2	80·2	72·4, 88·1	0·848
Unfavourable foods‡	70·4	56·5, 84·6	76·2	71·0, 81·5	65·1	54·7, 75·4	0·274	74·3	58·6, 89·9	77·8	72·5, 83·0	80·0	72·5, 87·5	0·775

* Weighted figures except *n* (= unweighted sample number), number of adolescents with missing SES: *n* 19.

† Significant differences between groups were tested using the *χ*^2^ test.

‡ Sweets, pastries, sugar-sweetened beverages.

Table 7 shows the mean consumption of foods by region of residence. Among girls from former West Germany, the mean consumption of total beverages was significantly higher than among girls from former East Germany (1768 ml *v*. 1517 ml; *P* < 0·01). Considering the specific beverages, we observed that compared with girls from former East Germany, girls from former West Germany had a higher mean consumption of water (1215 ml *v*. 814 ml; *P* < 0·001) but a lower mean consumption of tea (133 ml *v*. 247 ml; *P* < 0·05). In addition, girls from former West Germany had a higher mean consumption of grain, pasta, rice (85 g *v*. 68 g; *P* < 0·001), vegetables (214 g *v*. 179 g; *P* < 0·01) and meat/meat products (90 g *v*. 74 g; *P* < 0·01) and a lower consumption of breakfast cereals (11 g *v*. 16 g; *P* < 0·01) than girls from former East Germany. Among boys from former West Germany, the consumption of oils and fat was lower compared with counterparts from former East Germany (37 g *v*. 29 g; *P* < 0·01).

**Table 7:** Mean consumption and 95 % CI of foods (g/d) among adolescents from Eating Study as a KiGGS Module (EsKiMo) II by sex and region of residence (*n* 1353)*

	Girls (*n* 727)					Boys (*n* 626)				
	Region of residence					Region of residence				
	Former East Germany (*n* 249)		Former West Germany (*n* 478)			Former East Germany (*n* 216)		Former West Germany (*n* 410)		
	Mean	95 % CI	Mean	95 % CI	*P* †	Mean	95 % CI	Mean	95 % CI	*P* †
Total foods (except beverages)	1097	1032, 1163	1123	1070, 1177	0·534	1369	1264, 1473	1334	1273, 1396	0·569
Bread, savoury baked goods	126	115, 137	137	127, 147	0·163	160	145, 176	159	149, 170	0·919
Grain, pasta, rice	68	63, 74	85	78, 92	0·000	98	74, 123	104	94, 113	0·687
Breakfast cereals	16	13, 19	11	8, 13	0·009	22	15, 28	21	14, 27	0·861
Salty snacks	5	3, 7	6	5, 8	0·337	6	4, 7	7	5, 8	0·328
Vegetables	179	162, 196	214	195, 233	0·007	176	142, 210	179	162, 195	0·889
Fruits	184	161, 208	158	144, 172	0·053	162	135, 189	131	114, 149	0·056
Potatoes	68	59, 76	65	58, 71	0·544	87	76, 98	82	72, 92	0·507
Eggs	14	12, 16	16	14, 17	0·275	23	18, 29	21	18, 24	0·482
Fats and oils	24	21, 26	24	23, 26	0·700	37	32, 41	29	26, 31	0·002
Milk/dairy products, cheese, curd	220	188, 252	197	177, 217	0·222	305	256, 355	320	281, 360	0·633
Meat/meat products	74	67, 80	90	81, 99	0·004	131	117, 144	130	119, 140	0·898
Fish	8	6, 9	9	7, 10	0·330	10	8, 12	13	10, 16	0·082
Confectionery, cake	93	82, 105	90	80, 99	0·644	126	110, 142	110	101, 119	0·079
Total beverages	1517	1398, 1636	1768	1670, 1866	0·001	2050	1856, 2244	2032	1924, 2140	0·869
Tea	247	141, 353	133	103, 164	0·039	104	69, 139	77	55, 99	0·186
Coffee	25	5, 45	26	16, 35	0·948	43	21, 65	26	18, 33	0·130
Juice	149	112, 187	182	147, 218	0·206	249	185, 314	242	200, 283	0·839
Soft drinks	262	201, 322	190	147, 232	0·052	420	319, 522	339	275, 402	0·173
Alcoholic beverages	20	12, 28	22	9, 36	0·772	49	26, 71	45	32, 59	0·806
Water	814	706, 921	1215	1114, 1316	< 0·001	1185	989, 1381	1304	1196, 1411	0·287

* Weighted figures except *n* (= unweighted sample number).

† Significant differences between groups were tested using ANOVA.

There were differences in food consumption regarding the proportion of adolescents achieving or exceeding the recommended amounts of the OMD according to the region of residence. Girls from former West Germany achieved the recommended amount for vegetables significantly more often than girls from former East Germany (vegetables: 18·6% *v*. 10·4%; *P* < 0·05). The recommended amount for total beverages was met significantly more often by girls from former West Germany (81·9 %) compared with girls from former East Germany (71·7 %; *P* < 0·05). The recommended amounts for oil/margarine/butter were achieved more often among boys from former East Germany (38·8 %) than among boys from former West Germany (23·8 %; *P* < 0·01) (Table 8).

**Table 8:** Proportion of adolescents from Eating Study as a KiGGS Module (EsKiMo) II achieving the Optimized Mixed Diet (OMD)-recommended amount by sex and region of residence (*n* 1353)*

	Girls (*n* 727)					Boys (*n* 626)				
	Region of residence					Region of residence				
	Former East Germany (*n* 249)		Former West Germany (*n* 478)			Former East Germany (*n* 216)		Former West Germany (*n* 410)		
	%	95 % CI	%	95 % CI	*P* †	%	95 % CI	%	95 % CI	*P* †
Fruits	16·3	10·2, 22·3	15·3	11·3, 19·3	0·787	5·6	2·2, 9·0	6·5	3·5, 9·5	0·704
Vegetables	10·4	6·1, 14·6	18·6	14·3, 22·9	0·011	7·9	3·7, 12·7	7·1	4·2, 10·1	0·761
Starchy food	16·1	10·3, 21·9	22·0	17·1, 26·9	0·141	21·7	14·9, 28·5	19·5	15·2, 23·9	0·591
Meat/meat products	69·6	62·9, 76·4	64·9	59·2, 70·6	0·299	79·6	72·4, 86·8	83·4	78·7, 88·1	0·371
Fish	18·8	12·6, 25·0	20·4	15·8, 25·0	0·687	22·7	15·8, 29·5	30·4	25·1, 35·6	0·089
Eggs	18·9	12·9, 25·0	22·8	17·8, 27·8	0·334	28·5	19·5, 37·5	25·0	18·6, 31·5	0·537
Milk/dairy products	18·2	11·5, 24·9	15·7	11·3, 20·1	0·534	18·0	11·7, 24·3	19·1	13·9, 24·4	0·783
Oil/margarine/butter	28·3	21·7, 35·1	28·9	24·2, 33·7	0·885	38·8	30·5, 47·2	23·8	19·2, 28·3	0·001
Beverages	71·7	65·4, 78·0	81·9	77·1, 86·8	0·011	77·0	68·9, 85·1	84·2	79·6, 88·7	0·108
Unfavourable foods‡	75·6	70·8, 80·5	71·9	66·2, 77·5	0·315	77·9	68·4, 87·4	78·1	73·1, 83·0	0·970

* Weighted figures except *n* (= unweighted sample number).

† Significant differences between groups were tested using the *χ*^2^ test.

‡ Sweets, pastries, sugar-sweetened beverages.

## Discussion

While the majority of adolescents in Germany consumed sufficient amounts of beverages, they did not achieve the recommended amounts of the OMD for most food groups. In particular, the consumption of fruits, vegetables, starchy foods and milk/dairy products of most adolescents aged 12–17 years in Germany was below the recommended amounts. The consumption of meat/meat products and unfavourable foods like sweets, pastries and sugar-sweetened beverages, which should be consumed sparingly, exceeded the recommended amounts. Though the total beverage consumption of the majority of adolescents was above the minimum amount recommended, a large part was reached through juices and soft drinks. Differences in food group consumption were also observed according to SES and region of residence; adolescents with a low SES had a higher soft drink consumption than those with a high SES. Girls from former West Germany more often achieved the recommended amount of vegetables compared with girls from former East Germany.

Our results are largely in line with results from the HELENA (Healthy Lifestyle in Europe by Nutrition in Adolescence) Study of a sample of European adolescents aged from 12 to 17 years^(26)^. For most food groups, the deviations from the recommended amounts of the OMD observed in HELENA are in a similar range and the same direction as the ones observed in EsKiMo II. There seems to be a clear discrepancy between HELENA and EsKiMo II only for total beverage consumption, with a higher proportion of adolescents achieving the recommended amount for beverages in EsKiMo II compared with HELENA. This can be explained by differences in food group classification between both studies. In HELENA, only water, tea and coffee were assigned to the food group beverages, whereas in EsKiMo II, fruit and vegetable juices as well as soft and alcoholic drinks were also included.

Both the HELENA Study^(26)^ and EsKiMo II observed that the consumption of fruit and vegetables of adolescents was clearly below the recommended amount of the OMD. In other European countries, children and adolescents also did not reach the recommendation for fruit and vegetable consumption^(26,27)^. The WHO recommends a daily consumption of 400 g of fruit and vegetables for adolescents^(28)^, which is lower than the recommended amount of the OMD. However, the mean consumption of 371 g/d among girls is almost in accordance with the WHO recommendation. According to the ranking of the Health Behaviour in School-aged Children (HBSC) study for daily fruit consumption in Europe, the fruit consumption among adolescents living in Germany is at midrange, whereas countries like Greenland and Finland are at the bottom and Armenia and Albania are at the top of this ranking^(29)^. An improvement of fruit and vegetable consumption could be achieved by consuming one additional portion of fruit and one additional portion of vegetables daily.

In EsKiMo II, only about 20 % of the adolescents reached the recommended amount for starchy foods. Shortcomings in the consumption of starchy foods of adolescents were also observed previously^(26)^.

Low consumptions of milk and dairy products by adolescents have been published previously^(26)^. The observation that even <25 % of the adolescents achieved the recommended amount for milk/dairy products indicates a need for action, especially because preliminary analysis of the nutrient intakes from EsKiMo II showed that Ca intake of the majority of adolescents is below the German nutrient recommendations (D-A-CH reference values) (unpublished results). Since milk and dairy products are an important Ca source^(30)^, a major cause of the deficits in Ca intake among adolescents may be the low consumption of milk and dairy products.

The median consumption of meat/meat products of adolescents in EsKiMo II was lower, as observed among adolescents in the HELENA Study^(26)^, but far above the recommended amount. A reduction of meat consumption is highly recommended. Current social developments and public discussions about vegetarianism, bio-industry, animal welfare, climate impact of meat consumption and feeding the increasing world population may lead to a reduction of meat consumption in the near future^(31)^.

The recommended amount of unfavourable food was also largely exceeded. An excessive consumption of energy-dense, nutrient-poor food is also seen in other European studies among adolescents^(26,32)^. As this food group mainly consists of foods with a high energy density and a low nutritive value, a reduction in the consumption of these foods or rather a shift of consumption from unfavourable to more favourable food groups like fruits, vegetables and unsweetened beverages is urgently needed.

Next to a comparison of the results from EsKiMo II and in the view of actual literature on food consumption of adolescents, a comparison with the results from EsKiMo I (2006)^(33)^ is possible. This comparison indicates some changes in food consumption between 2006 and 2015–2017; the proportion of adolescents who achieved the recommended amounts of fruits, vegetables and milk/dairy products decreased over time. However, the proportion of adolescents who achieved the recommended amount of starchy foods has increased. The proportion of the recommended amount achieved for beverages, unfavourable foods and fish was similar in EsKiMo I and EsKiMo II. However, it should be considered that the recommended amounts of the OMD for the food groups fruits, vegetables, milk/dairy products and starchy foods have been changed between 2006 and 2015^(20)^. Additional analysis evaluating the food group consumption within EsKiMo I according to the actual OMD recommendations also shows a decrease in the proportion of adolescents achieving the recommended amount for fruits and milk/dairy products between both EsKiMo studies, and also the proportion of adolescents achieving the recommended amount of starchy foods decreased when using recalculated data for EsKiMo I (data not shown).

In both EsKiMo I^(33)^ and EsKiMo II, sex differences in food consumption and in adherence to the food-based dietary guidelines have been observed. In both studies, absolute food consumption was higher among boys, conforming to the mainly higher requirements for boys than for girls. However, compared with girls, a lower proportion of boys achieved the recommended amounts of fruit and vegetables. Furthermore, boys showed a higher consumption of soft drinks. Generally, a poorer dietary quality among boys has already been reported in several studies conducted among adolescents and young adults in Belgium, New Zealand and the USA^(32,34,35)^. Reasons for the differences between boys and girls might be differences in preferences, amongst others. Girls have a higher affinity for fruits and vegetables and perceive a lower barrier to their consumption^(36)^. Sex differences in fruit and vegetable consumption were also seen among adults living in Germany^(37,38)^.

The results of EsKiMo II indicate a poorer dietary quality for adolescents with a low SES. The strongest social gradient is seen for soft drinks; boys and girls with a low SES have a significant higher consumption of soft drinks than adolescents with a high SES. This is in line with previous studies among adolescents, for example, the KiGGS Wave 1 and HBSC studies conducted in Germany and European Countries, respectively^(11,12,39)^. Girls with a low SES have a significantly higher consumption of meat/meat products compared with girls with a high SES. A similar association between meat consumption and SES has already been reported for other populations^(40)^. However, the association between SES and the consumption of fruit^(8,9)^ and vegetables^(8–10)^ observed in previous studies, also among adults^(37,38)^, is not seen in EsKiMo II. This may be due to projects focused on the improvement of fruit and vegetable consumption, such as the EU school fruit and vegetable schemes that reach adolescents from all social backgrounds or projects focusing on socially disadvantaged adolescents conducted in the last decade^(41)^.

To our knowledge, this is the first investigation of food consumption including a comparison with food-based dietary guidelines among adolescents by region of residence in Germany. Several differences in consumption were observed for former East and West Germany. These differences are especially seen among girls. One explanation for these differences may be that former East Germany has more rural regions, a different SES structure and a longer tradition of school meal supply and a higher utilisation of school meals^(42)^, which may contribute to the observed differences and may have a larger impact among girls. In Germany, the availability and usage of school meals have grown in recent years. In addition, there is a programme to improve the quality of these meals^(43)^. This development may contribute to preferable changes in the diet of adolescents.

This study has strengths and limitations. A limitation is that information on food consumption was collected retrospectively and based on self-reports, which might lead to recall bias. Also, particular foods may be under-reported (e.g. confectionery, sugar-sweetened beverages) or over-reported (e.g. fruits and vegetables) due to social desirability. As a consequence, the deficits of food consumption may be larger. In addition, the assignment of food groups is very complex as composed foods can be included as such in a food group, that is, bread and cakes, while other composed foods, which cannot be assigned to a single food group, were decomposed to their ingredients, being assigned into the corresponding food groups. Although this process was highly standardised, conducted by a nutritionist and double-checked, some level of misclassification of single foods cannot be excluded. The consumption of fruits might be slightly higher than reported, as cakes were not decomposed into food groups. In case of disaggregating particular cakes containing fruits into their ingredients, there might be a shift in the allocation of foods to food groups from unfavourable foods to fruits. There are also some restrictions in comparison with the OMD amounts. The definition of food groups of the OMD is relatively broad and lacks some details about which particular foods should be included or excluded. Although the OMD does not specifically mention this, we assumed that the recommended amounts of the OMD refer to amounts of foods as consumed (not as raw quantities). These amounts were used as the basis for our calculations. In addition, the inclusion of sugar-sweetened beverages in the total amount of beverages as well as in the food group of the unfavourable foods is ambiguous. So, the consumption of fruit and vegetables expressed as proportion of recommended amounts may be underestimated, and evaluation of total beverage consumption does not differentiate between the contribution of preferable and non-preferable beverages. This study gives an overview of the recent food intake of adolescents in Germany and its distribution in subgroups. It is mainly a descriptive analysis without intending to detect causal relationships or to test *a priori* hypotheses. Nevertheless, many *P*-values are presented, and the implication of multiple testing should be considered. However, for this explorative situation using variables which are not completely independent (food groups as well as SES/region), a correction for multiple testing may be difficult and not appropriate, as suggested in literature^(44,45)^. Nevertheless, the significance of results should be interpreted with caution. The strength of the study is that it was conducted nationwide with a large representative sample and reflects the food consumption of adolescents in Germany.

The results underline the need for interventions that improve the food consumption of adolescents, especially to accomplish the shift from a high consumption of unfavourable foods and meat to a higher consumption of fruit, vegetables, foods with complex carbohydrates and milk/dairy products or adequate substitutes. This is a major challenge for all public health professionals. In 2008, the national action plan ‘IN FORM – German national initiative to promote healthy diets and physical activity’ was launched with the aim to promote a healthy lifestyle with a balanced diet and sufficient exercise in all living environments. Already developed initiatives and strategies from federal and state governments and municipalities were complemented and deepened. Most measures, projects or recommendations take place in the living environment as a way to make changes in behaviour easier in everyday life. For instance, projects in schools can reach the whole generation of young people of all social levels^(41)^. However, the observed differences in food consumption between EsKiMo I and II indicate that the existing measures may have led to only limited improvements in preferable food consumption so far. Further approaches to promote a healthy diet, such as easy to understand front-of-package labelling, a restriction on the promotion of unhealthy foods through all media and price policies for healthy and unhealthy foods, should be considered.

In conclusion, the current overview of the food consumption of adolescents in Germany shows that the consumption of fruit and vegetables is far below the recommendation, whereas the consumption of meat and unfavourable foods, like sugar-sweetened beverages and confectionery, is too high. Social disparities are seen in the consumption of soft drinks. This indicates that approaches to promote healthy diets should be continued and the focus on social inequalities should be strengthened.
